# Controlling the thermal conductivity of multilayer graphene by strain

**DOI:** 10.1038/s41598-021-98974-x

**Published:** 2021-10-01

**Authors:** Kaito Nakagawa, Kazuo Satoh, Shuichi Murakami, Kuniharu Takei, Seiji Akita, Takayuki Arie

**Affiliations:** 1 Department of Physics and Electronics, Osaka Prefecture University, 1-1 Gakuencho, Naka-ku, Sakai, Osaka 599-8531 Japan; 2 Osaka Research Institute of Industrial Science and Technology, 2-7-1 Ayumino, Izumi, Osaka 594-1157 Japan

**Keywords:** Mechanical and structural properties and devices, Mechanical and structural properties and devices, Two-dimensional materials

## Abstract

Straintronics is a new concept to enhance electronic device performances by strain for next-generation information sensors and energy-saving technologies. The lattice deformation in graphene can modulate the thermal conductivity because phonons are the main heat carriers. However, the device fabrication process affects graphene’s heat transport properties due to its high stretchability. This study experimentally investigates the change in the thermal conductivity when biaxial tensile strain is applied to graphene. To eliminate non-strain factors, two mechanisms are considered: pressure-induced and electrostatic attraction–induced strain. Raman spectroscopy and atomic force microscopy precisely estimate the strain. The thermal conductivity of graphene decreases by approximately 70% with a strain of only 0.1%. Such thermal conductivity controllability paves the way for applying graphene as high-efficiency thermal switches and diodes in future thermal management devices.

## Introduction

For effective thermal management and energy recycling of devices, understanding the thermal transport properties of materials is crucial. A heat spreader requires a high thermal conductivity material to dissipate the heat generated from electronic devices efficiently. On the other hand, thermoelectric conversion has attracted much attention as a renewable energy. This technology requires materials with higher electrical but lower thermal conductivities. Consequently, many theoretical and experimental studies have investigated the reduction in thermal conductivity to improve the thermoelectric conversion efficiency.

Two-dimensional (2D) materials composed of one-atom-thick layers exhibit outstanding characteristics, which differ from those of three-dimensional materials, and are expected to realize future electronic devices^1^. As a representative 2D material, graphene has potential for mechanical^2–4^ and electrical^5–8^ devices. Graphene displays much higher thermal transport properties than existing materials^9–12^. Due to its 2D nature, its thermal conductivity can easily be modified by introducing carbon isotopes^13–15^, structural defects^16–19^, and domain boundaries^20,21^. These modifications can control the thermal conductivity in applications such as thermal management devices.

Since phonons are the main heat carriers in graphene, strain-induced lattice deformation easily modulates phonon propagation, leading to changes in the thermal conductivity. Due to its high stretchability, the device fabrication process affects graphene’s heat transport properties. To utilize graphene in electronic and thermal management devices, elucidating its thermal transport properties against strain is essential. Although the thermal conductivity of graphene with strain was recently reported^22^, the change in its thermal conductivity due to the continuous introduction of strain has yet to be elucidated. In this study, we investigate the thermal conductivity changes due to strain in identical graphene drums. To eliminate other factors that may alter the thermal conductivity, strain was introduced by two different mechanisms: pressure difference–induced strain (DEVICE 1) and electrostatic attraction–induced strain (DEVICE 2). The thermal conductivity was measured using Raman spectroscopy, which is very sensitive to the number of layers^23–25^, temperature^26,27^, and strain^28,29^. AFM-based analyses were used to precisely estimate the strain into graphene.

## Device fabrication

**Figure 1:**
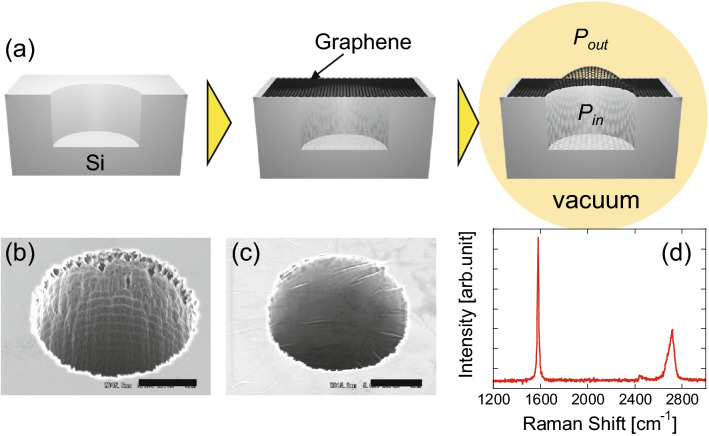
(**a**) Cross-sectional illustration of introduced strain by the pressure difference in the device structure for DEVICE 1. Scanning electron micrographs of the hole fabricated in the substrate (**b**) before and (**c**) after graphene transfer. Bars represent 5 μm. (**d**) Typical Raman spectrum of graphene used for DEVICE 1, showing multilayer graphene with negligible defects.

**Figure 2:**
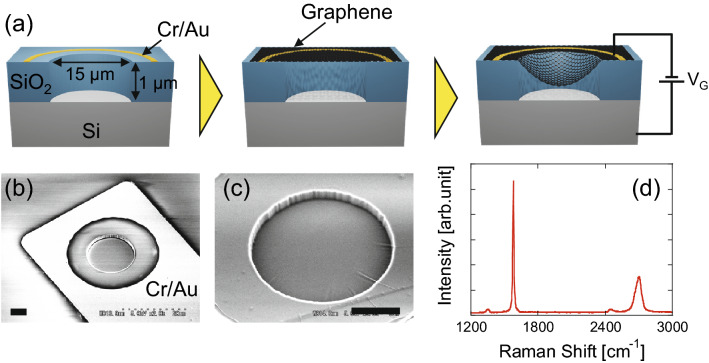
(**a**) Cross-sectional illustration of introduced strain by the electrostatic attraction in the device structure for DEVICE 2. Scanning electron micrographs of the hole fabricated in the substrate (**b**) before and (**c**) after graphene transfer. Bars represent 5 μm. (**d**) Typical Raman spectrum of graphene used for DEVICE 2, showing multilayer graphene with negligible defects.

We fabricated two types of graphene drum structures. DEVICE 1 controls the strain application by the pressure difference (Fig. 1), whereas DEVICE 2 uses electrostatic force (Fig. 2). To fabricate DEVICE 1, we first formed cylindrical holes with a 15-μm diameter in the Si substrate via a chromium mask using photolithography and deep reactive ion etching with SF_6_ and C_4_F_8_ gases (MUC-21 ASE-SRE, SPP technologies). The holes with the depth of approximately 170 μm do not pass through the substrate (Fig. 1a). Single crystalline multilayer graphene was prepared by a mechanical exfoliation technique^30,31^. Specifically, we exfoliated graphene flakes from kish graphite using scotch tape. The tape was then placed on a PDMS gel to transfer the graphene flakes. Subsequently, we put the PDMS gel on a Si substrate at atmospheric pressure to transfer graphene onto the hole and produce a suspended drum-shaped graphene membrane. Because the pressure inside the hole P_in_ remains at atmospheric pressure P_atm_ due to the impermeability of graphene to air^32^, the pressure difference between inside and outside of the hole results in a graphene bulge when the substrate is placed in a vacuum ($P_{out} < P_{atm}$) (Fig. 1a).

Figure 1b,c show scanning electron micrographs of the hole before and after graphene transfer, respectively. The bulge deformation induces biaxial strain into the graphene membrane. The strain can be controlled by the pressure outside the hole P_out_. The Raman spectrum of the graphene after transfer (Fig. 1d) indicates that the graphene is a single-crystalline multilayer with negligible structural defects^23–25,33^.

To fabricate DEVICE 2, 15-μm-wide and 1-μm-deep cylindrical holes were formed via photolithography and reactive ion etching with CF_4_ and CH_2_F_2_ gases (NLD-800, ULVAC) in a Si/SiO_2_ substrate on which the Cr/Au (20 nm/80 nm) electrodes were deposited in advance. The graphene film, which was mechanically exfoliated from kish graphite, was transferred to produce a suspended drum-shaped graphene membrane as described previously. Strain was applied by the electrostatic force generated with the electrical voltage V_G_ between graphene and the Si substrate (Fig. 2a). Figure 2b,c show the fabricated device structure before and after graphene transfer, respectively. A few wrinkles appear at the edge of the drum (Fig. 2c), indicating that the hole is completely covered by a graphene membrane. The Raman spectrum of the graphene indicates that the graphene is a single-crystalline multilayer membrane with negligible structural defects.

## Results and discussion

### Pressure-induced mechanical deformation of graphene in DEVICE 1

**Figure 3:**
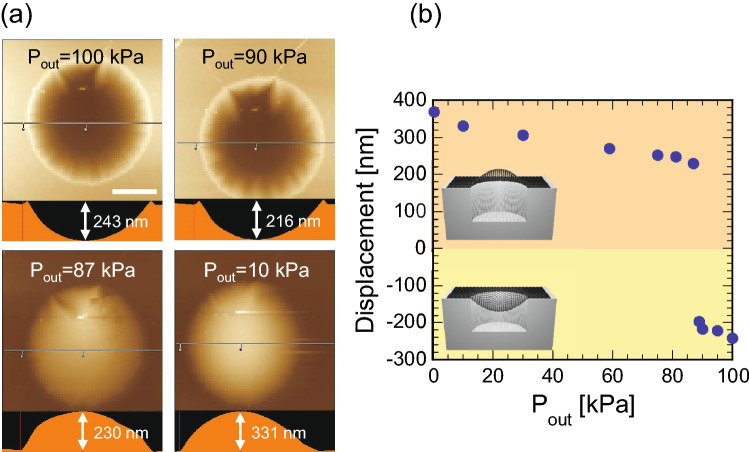
(**a**) Top and cross-sectional views of AFM images of the graphene drum for DEVICE 1 at various pressures P_out_. Bar in the image is 5 μm. (**b**) Displacement of the graphene drum at the various P_out_, showing the discontinuity from the downward to the upward deflection.

Atomic force microscopy (AFM) was used to observe the surface morphology of the graphene bulge at various pressures (Fig. 3). The samples were set in a vacuum chamber equipped with AFM (SPA-300HV, HITACHI), where the pressure in the chamber was changed with a variable leak bulb. A cantilever with a spring constant of 2 N/m was used in a dynamic force mode, in which the probe oscillates and intermittently touches the surface to minimize the deformation of the sample. Once reaching the appropriate pressure, AFM images were collected with the vacuum pump turned off to avoid the vibration problem. Figure 3a shows the top and cross-sectional images of the graphene drum at atmospheric pressure. The drum is initially deflected inward, possibly due to the mechanical pressure upon transferring the exfoliated graphene. As the background pressure in the vacuum chamber P_out_ decreases, graphene gradually begins to bulge and the center height of the drum increases (Fig. 3b). It should be noted that a displacement jump occurs around 90 kPa, implying that the graphene membrane exhibits slack and behaves similar to a traditional Japanese toy, vidro, when the displacement is 0. Nonetheless, the graphene drum monotonically bulges as the background pressure decreases after 87 kPa. A combination of Raman spectroscopy and AFM can estimate the mechanical strain induced into graphene.

### Thermal conductivity measurements of DEVICE 1

We measured the thermal conductivity of graphene using Raman thermometry^34^. The thermal conductivity *κ* is calculated by

1 $$ \kappa = \frac{{\alpha \times \ln \left( {{a \mathord{\left/ {\vphantom {a {r_{0} }}} \right. \kern-\nulldelimiterspace} {r_{0} }}} \right)}}{2\pi t} \cdot \frac{{Q_{abs} }}{\Delta T}, $$

where *a* is the radius of the graphene drum (7.5 μm), *r*_0_ is the radius of the laser beam, *t* is the graphene thickness, *Q*_*abs*_ is the laser power absorbed by graphene, and Δ*T* is the temperature rise induced by laser irradiation. In our setup, α is 0.98^19^. The graphene thickness estimated by AFM is 6.1 nm, which corresponds to 18 layers. Assuming that the temperature of the drum edge is at room temperature, the temperature rise Δ*T* at the center of the drum is estimated by^35^

2 $$ \Delta T = Q_{abs} \cdot {{\frac{{\partial \omega_{G} }}{{\partial Q_{abs} }}} \mathord{\left/ {\vphantom {{\frac{{\partial \omega_{G} }}{{\partial Q_{abs} }}} {\frac{{\partial \omega_{G} }}{\partial T}}}} \right. \kern-\nulldelimiterspace} {\frac{{\partial \omega_{G} }}{\partial T}}} = Q_{abs} \cdot \frac{{\partial \omega_{G} }}{{\partial Q_{abs} }} \cdot \chi^{ - 1} , $$

where $\chi = {{\partial \omega_{G} } \mathord{\left/ {\vphantom {{\partial \omega_{G} } {\partial T}}} \right. \kern-\nulldelimiterspace} {\partial T}}$ is the temperature coefficient of the Raman G band. From Eqs. (1) and (2), the thermal conductivity is derived as

3 $$ \kappa = \frac{{\alpha \times \ln \left( {{a \mathord{\left/ {\vphantom {a {r_{0} }}} \right. \kern-\nulldelimiterspace} {r_{0} }}} \right)}}{2\pi t} \cdot \chi \cdot \left( {\frac{{\partial \omega_{G} }}{{\partial Q_{abs} }}} \right)^{ - 1} . $$

χ and ${{\partial \omega_{G} } \mathord{\left/ {\vphantom {{\partial \omega_{G} } {\partial Q_{abs} }}} \right. \kern-\nulldelimiterspace} {\partial Q_{abs} }}$ are the slopes of the temperature-dependent and absorbed laser power–dependent G band peak shifts, respectively. Although the experimentally obtained Raman spectra can calculate these differentials, the strain cannot be controlled for graphene supported on the substrate. Herein the temperature coefficient of the G band, ${{\partial \omega_{G} } \mathord{\left/ {\vphantom {{\partial \omega_{G} } {\partial T}}} \right. \kern-\nulldelimiterspace} {\partial T}}$, is assumed to be constant and independent of the induced strain. Hence, the thermal conductivity measurement is used to determine the change in the laser power-dependent G band shift due to strain, ${{\partial \omega_{G} } \mathord{\left/ {\vphantom {{\partial \omega_{G} } {\partial Q_{abs} }}} \right. \kern-\nulldelimiterspace} {\partial Q_{abs} }}$.

**Figure 4:**
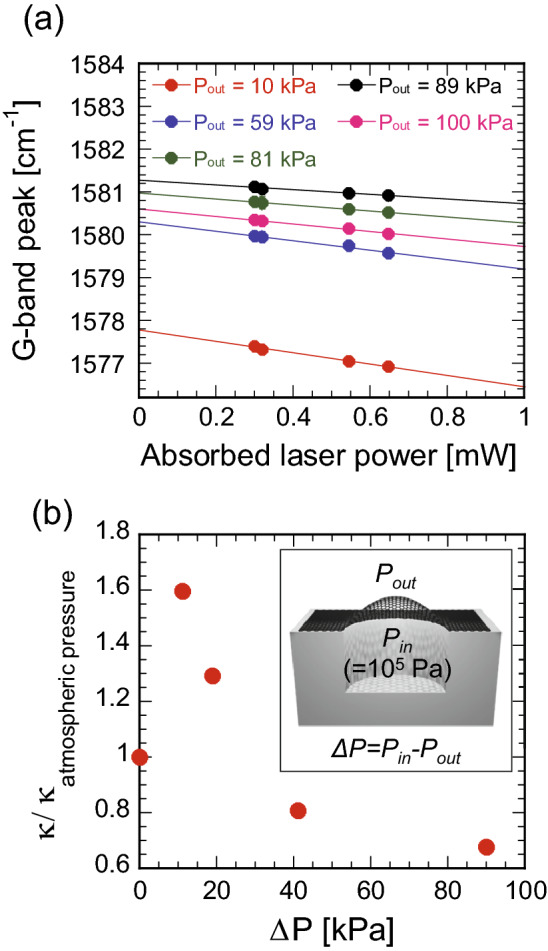
(**a**) Raman G band peak positions of the graphene drum (DEVICE 1) obtained with four different laser powers (0.71, 0.75, 1.27, and 1.52 mW) at various pressures P_out_. (**b**) Change in the thermal conductivity with respect to the pressure difference $\Delta {\text{P}} = P_{in} - P_{out}$. Values are normalized by that at atmospheric pressure.

Raman spectra were measured at four different laser powers (0.71, 0.75, 1.27, and 1.52 mW) to calculate ${{\partial \omega_{G} } \mathord{\left/ {\vphantom {{\partial \omega_{G} } {\partial Q_{abs} }}} \right. \kern-\nulldelimiterspace} {\partial Q_{abs} }}$ at various pressure P_out_. The samples were set in a vacuum chamber (THMS350V, Linkam Scientific), where the pressure in the chamber was changed with a variable leak bulb. After reaching the appropriate pressure, Raman spectra were collected with the vacuum pump turned off to avoid the vibration problem. Raman spectra were obtained with a 100 × objective using a 532-nm laser as an excitation source. The Raman line scanning mode across a steep edge of a gold electrode on Si/SiO_2_ substrate estimated the diameter is 0.65 μm^36^. Figure 4a shows the absorbed laser power dependence of the G band peak position. Normally, we collected Raman spectra 8–10 times with the same laser power at the same position and averaged the wavenumber values to minimize uncertainty of the measurement. The G band position normally shifts to lower wavenumbers as P_out_ decreases due to the induced strain, except at 100 kPa, where the graphene drum is initially bent inward (Fig. 3). From the linear fitting, ${{\partial \omega_{G} } \mathord{\left/ {\vphantom {{\partial \omega_{G} } {\partial Q_{abs} }}} \right. \kern-\nulldelimiterspace} {\partial Q_{abs} }}$ at various P_out_ can be derived. Using Eq. 3, the thermal conductivity changes with respect to $\Delta {\text{P}} = P_{in} - P_{out}$ can be determined (Fig. 4b). Changing the pressure monotonically changes the convective heat transfer. Therefore, the change in the thermal conductivity is primarily due to the strain induced by the pressure difference and not the convection. The reduced thermal conductivity at atmospheric pressure is probably because the graphene drum is bent inward, causing initial tension on the graphene membrane. Note that the temperature rise at the center of graphene is only in the range of 10 °C in our experiment. Based on the temperature rise, the radiation power estimated by Stefan–Boltzmann law is only four orders of magnitude smaller than the laser power absorbed into graphene, which is negligible in our thermal conductivity measurement.

### Strain by the pressure difference in DEVICE 1

To estimate the strain induced by the pressure difference, AFM measured the mechanical deformation of the graphene membrane as P_out_ was changed. Then an analytical model^37^ estimated the stress and strain induced at the center of the membrane when the circular graphene drum bulged. In this model, in-plane stress (*σ*) and in-plane strain $\varepsilon_{AFM}$ derived by AFM are respectively expressed by

4 $$ \sigma = \frac{{\Delta Pa^{2} }}{4ht} $$

5 $$ \varepsilon_{AFM} = \frac{{2\left( {h^{2} - h_{0}^{2} } \right)}}{{3a^{2} }}. $$

Δ*P* is the pressure applied to graphene $\Delta P = P_{in} - P_{out}$. *a* is the radius of the graphene membrane (7.5 μm). *t* is the graphene membrane thickness (6.1 nm). *h* is the height of the bulge measured by AFM, and *h*_0_ is the height of the membrane at a strain of 0. This model can be applied when the membrane is initially slack. From the above equations, stress $\sigma$ and strain $\varepsilon_{AFM}$ can be used to evaluate the mechanical strength of graphene from the AFM measurements.

From Fig. 3b, assuming that $h_{0} \approx 230$ nm when P_out_ is 87 kPa, the in-plane stress and strain induced into graphene can be calculated at a given pressure by Eqs. (4) and (5). Figures S1a and S1b show the strain–pressure $\varepsilon_{AFM}$–P_out_ and stress–strain $\sigma$–$\varepsilon_{AFM}$ curves of the graphene drum based on the AFM measurements, respectively. Based on the linear relationship between stress and strain, we calculated Young’s modulus as 0.78 TPa. This value is consistent with the simulation results^38^, in which Young’s modulus of multilayer graphene tends to decrease as the number of layers increases^2^.

Then we extracted the change in the Raman peaks due to strain from Fig. 4a. In general, the Raman peak position shifts to lower wave numbers when a tensile strain is applied to graphene^28,29^. Since the Raman peak positions are temperature-dependent^26,27^, the effect of laser heating on the Raman spectra cannot be ignored. Therefore, we extrapolated the G peak position without the laser heating effect from the y-intercept in Fig. 4a.

Finally, by combining the strain estimated from AFM $\varepsilon_{AFM}$ with the y-intercept values of the G peak position obtained from Fig. 4a at the same P_out_, the strain induced into graphene can be estimated more precisely. Figure S1c shows the relationship between the estimated strain and the intercept values in Fig. 4a. The Raman peak shift, which depends on the strain as $\partial \omega_{G} /\partial \varepsilon_{AFM} \approx - 56.8\,[{\text{cm}}^{ - 1} /\% ]$, was calculated using a linear fitting. The intercept ω_G0_ is 1581.6 cm^-1^. This coincides with the experimentally obtained G band position without the strain effect. Hence, the strain can be estimated from the Raman spectra $\varepsilon_{Ram}$ using the following equation

6 $$ \varepsilon_{Ram} = \left( {\frac{{\partial \omega_{G} }}{{\partial \varepsilon_{AFM} }}} \right)^{ - 1} \times \left( {\omega_{G} - \omega_{G0} } \right). $$

Figure S1d depicts the stress–strain curve obtained from the Raman spectra during the thermal conductivity measurements (Fig. 4a). The estimated Young’s modulus is 0.78 TPa, which is almost consistent with the results obtained by AFM. Table 1 summarizes the strain actually induced into graphene in the thermal conductivity measurement (Fig. 4b).

**Table 1:** Summary of the estimated strain values at various P_out_ for DEVICE 1.

P_out_ [kPa]	ΔP [kPa]	ω_G_ [cm^−1^]	Δω_G_ [cm^−1^]	ε [%]
10	90	1577.8	− 3.8	0.068
59	41	1580.3	− 1.3	0.023
81	19	1581.0	− 0.6	0.011
89	11	1581.3	− 0.3	0.006
100	0	1580.6	− 1.0	0.018

### Mechanical deformation of graphene by electrostatic attraction in DEVICE 2

**Figure 5:**
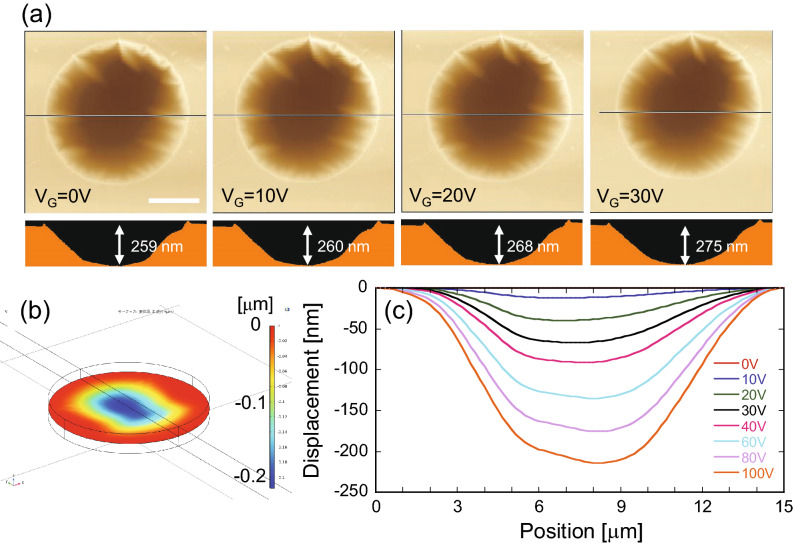
(**a**) Top and cross-sectional views of AFM images of the graphene drum for DEVICE 2 at various applied voltages V_G_. (**b**) Model of the graphene drum used for the finite element calculation (COMSOL) merged with the calculated displacement at V_G_ = 100 V. (**c**) FEM results of the displacement as a function of the drum position at various voltages.

In DEVICE 2, the strain was induced by electrostatic attraction, which was generated by applying voltage V_G_ between graphene and the Si substrate. Figure 5a shows a series of AFM images at various V_G_. The displacement of the graphene drum increases with increasing V_G_. The trend is consistent with the finite element calculation (COMSOL) with a graphene thickness of 3.4 nm and Young’s modulus of 0.78 TPa (Fig. 5b). The discrepancy in the displacement between the AFM results and the calculations (Fig. 5c) may be because the graphene drum is already deflected inward when V_G_ = 0 V due to the pressure during the mechanical transfer process, as mentioned in DEVICE 1. Thus, the strain induced into graphene by applying V_G_ must be precisely identified during thermal conductivity measurements.

### Thermal conductivity measurement of DEVICE 2 by Raman spectroscopy

**Figure 6:**
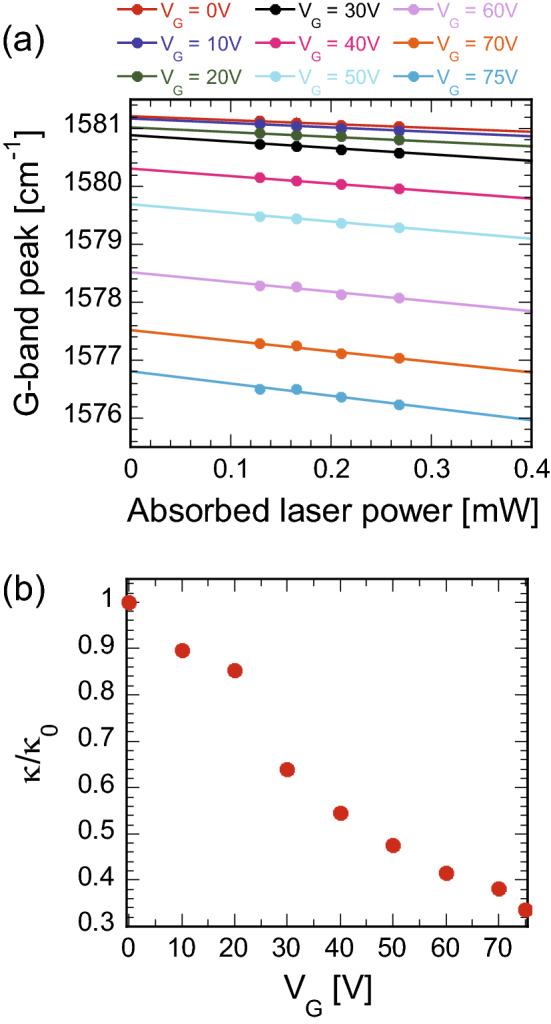
(**a**) Raman G band peak positions of the suspended graphene drum for DEVICE 2 obtained with four different laser powers at various voltages V_G_. (**b**) Change in the thermal conductivity with respect to the voltage. As V_G_ increases, voltage dramatically decreases. Values are normalized by that at V_G_ = 0 V.

We calculated the thermal conductivity of DEVICE 2 at various V_G_ using Eq. (3). The procedure was the same as that for DEVICE 1. The estimated graphene for DEVICE 2 by AFM is 3.4 nm, which corresponds to 10 layers. Figure 6a shows the absorbed laser power dependence of the Raman G band when V_G_ is applied to deflect the graphene drum downward. Figure 6b shows the calculated thermal conductivity of the graphene drum at various V_G_ using the linear fitting to the data. Each value was normalized by the thermal conductivity at V_G_ = 0 V. The thermal conductivity dramatically decreases by approximately 70% when V_G_ = 75 V. Next, we converted the voltage-dependent deflection into strain to elucidate the effect of the strain on the thermal conductivity.

### Strain by electrostatic attraction in DEVICE 2

Both strain and charge doping shift the Raman G and 2D peaks^28,29,39–44^. To precisely identify the strain induced in graphene, the effects of strain must be separated from those of charge doping. Since strain- and doping-induced changes in the Raman G and 2D peaks are correlated by a linear relationship^40,44^, we used correlation analysis to separate the effect of strain from that of charge doping.

### Raman spectral shift by charge doping effect

To identify the Raman spectral shift due to charge doping, we measured the G and 2D peak positions of the Raman spectra from graphene supported on the substrate while changing the positive voltage V_G_ (electron doping) using the device shown in Fig. 2a. Figures S2a and S2b show the laser power dependence of the G peak and 2D peaks at various V_G_. As mentioned above, the y-intercepts were obtained as the values of G and 2D band peak positions without the laser heating effect. Consistent with a previous report^44^, the G and 2D peak positions change positively with respect to the applied voltage V_G_ (Fig. S2c). From the linear relationship between G and 2D peak positions (Fig. S2d), $\left. {\partial \omega_{2D} /\partial \omega_{G} } \right|_{doping} \approx 0.31$ for electron doping in our device.

### Raman spectral shift by the strain effect

We then measured the G and 2D peak positions of the Raman spectra using the graphene bulge device shown in Fig. 1a. By combining the Raman G and 2D peak positions with the AFM-based strain estimation used for DEVICE 1, we can identify the Raman peak shift due only to the strain effect. Because the relative change in the strain is required, Eq. (5) can be rewritten as

7 $$ \Delta \varepsilon = \frac{{2\left( {h_{1}^{2} - h_{2}^{2} } \right)}}{{3a^{2} }}, $$

where *h*_1_ and *h*_2_ are the heights of the graphene bulge at two different background pressures P_out_.

Figure S3a represents the top and cross-sectional AFM images when the pressure outside the hole P_out_ is 10, 30, 50, and 70 kPa. For a given P_out_, the laser power dependences of the G and 2D peak positions were obtained by Raman spectroscopy (Figs. S3b and S3c). Both the G and 2D peak positions change downward as P_out_ increases, indicating an increase in the induced strain into graphene. Hence, the strain-dependent G band peak shift is given as ${{\partial \omega_{G} } \mathord{\left/ {\vphantom {{\partial \omega_{G} } {\partial \varepsilon }}} \right. \kern-\nulldelimiterspace} {\partial \varepsilon }} \approx - 53.5\;[{\text{cm}}^{ - 1} /\% ]$ for our multilayer graphene (Fig. S3d). Finally, the slope of the linear correlation between the Raman G and 2D peak positions gives $\left. {{{\partial \omega_{2D} } \mathord{\left/ {\vphantom {{\partial \omega_{2D} } {\partial \omega_{G} }}} \right. \kern-\nulldelimiterspace} {\partial \omega_{G} }}} \right|_{strain} \approx 2.1$ (Fig. S3e), which agrees well with the reported value^40^. Both $\left. {{{\partial \omega_{2D} } \mathord{\left/ {\vphantom {{\partial \omega_{2D} } {\partial \omega_{G} }}} \right. \kern-\nulldelimiterspace} {\partial \omega_{G} }}} \right|_{doping}$ and $\left. {{{\partial \omega_{2D} } \mathord{\left/ {\vphantom {{\partial \omega_{2D} } {\partial \omega_{G} }}} \right. \kern-\nulldelimiterspace} {\partial \omega_{G} }}} \right|_{strain}$ are required for the vector decomposition of the charge doping and strain effects from Raman spectra, while ${{\partial \omega_{G} } \mathord{\left/ {\vphantom {{\partial \omega_{G} } {\partial \varepsilon }}} \right. \kern-\nulldelimiterspace} {\partial \varepsilon }}$ is used to extract the precise strain value from the Raman G band peak shift.

### Separation of effects due to charge doping and strain

The absorbed laser power dependence of the Raman G band peak was used for the thermal conductivity measurements (Fig. 6a). For the vector decomposition of charge doping and strain effects for the suspended graphene drum (DEVICE 2), both the G and 2D band peak positions with respect to the laser power (Figs. 6a and S4a) were used. Figure S4b depicts the values of y-intercepts extracted from Figs. 6a and S4a at various applied voltages V_G_. There is a downward relation to V_G_. G and 2D peaks shift toward lower wavenumbers due to both charge doping and strain effects, although charge doping causes an upward relation as V_G_ increases (Fig. S2c)^44^. To eliminate the charge doping effect and estimate the exact strain from the Raman spectra, we implemented a decomposition method based on a simple vector model^40^ (Fig. S4c), where each measured result was decomposed from the charge doping effect and the strain effect. By considering the coefficient (Δω_G_) of the strain vector (slope: 2.1) with ${{\partial \omega_{G} } \mathord{\left/ {\vphantom {{\partial \omega_{G} } {\partial \varepsilon }}} \right. \kern-\nulldelimiterspace} {\partial \varepsilon }} \approx - 53.5\;[{\text{cm}}^{ - 1} /\% ]$, all the strain at the various applied voltage V_G_ was estimated relative to that when V_G_ = 0 V (Table 2). The strain increases with increasing V_G_ and reaches a maximum value of approximately 0.1% at V_G_ = 75 V in DEVICE 2.

**Table 2:** Summary of the estimated strain values at various V_G_ for DEVICE 2.

V_G_ [V]	Δω_G_ [cm^−1^]	ε [%]
0	0	0
10	− 0.04	0.0008
20	− 0.20	0.0038
30	− 0.58	0.011
40	− 1.31	0.025
50	− 2.10	0.040
60	− 3.17	0.060
70	− 4.32	0.081
75	− 5.22	0.098

### Strain-dependent thermal conductivity changes

**Figure 7:**
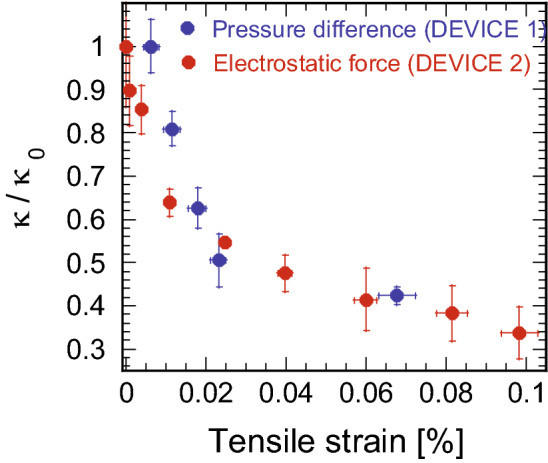
Thermal conductivity changes with respect to the strain induced into graphene for DEVICE 1 and DEVICE 2. Regardless of the strain introduction mechanism, the thermal conductivity decreases by approximately 60% with a strain of only 0.07%.

Finally, we converted pressure ΔP in Fig. 4b and voltage V_G_ in Fig. 6b into strain to elucidate the strain-induced thermal conductivity changes. Here, the thermal conductivity was calculated with the standard deviation obtained by propagation of error from Figs. 4a and 6a. Figure 7 depicts the thermal conductivity changes as a function of the tensile strain for both DEVICE 1 and DEVICE 2. They exhibit nearly identical trends, demonstrating that the thermal conductivity decreases by 70% when a tensile strain of approximately 0.1% is applied. Note that the absolute thermal conductivity values at minimum strain are 1848 ± 113 W/mK (DEVICE 1) and 4688 ± 655 W/mK (DEVICE 2), which are higher than the reported thermal conductivity values for bulk graphite^11^. Those high thermal conductivity values are due to the estimated high temperature coefficients, χ, of − 0.0165 cm^-1^/K (DEVICE 1) and − 0.0296 cm^-1^/K (DEVICE 2) in our experiment compared to the reported value (− 0.011 cm^-1^/K)^27,45^. The strain induced in graphene may vary depending on the temperature when measuring the temperature coefficient^46,47^, implying that the temperature coefficients became unexpectedly high. As expressed in Eq. (3), however, χ values do not affect the relative change in thermal conductivity when changing applied strain. Therefore, we discuss the strain-induced thermal conductivity change in multilayer graphene.

Since the deflection mechanisms differ between DEVICE 1 (pressure difference) and DEVICE 2 (electrostatic attraction), the change in the thermal conductivity primarily originates from the biaxial strain induced into the graphene network. It is noteworthy that the stain-induced thermal conductivity change is observed not only for multilayer graphene used in this study but also for exfoliated monolayer graphene^22^ as well as chemically grown monolayer graphene (data not shown). Thus, the thermal conductivity reduction by the strain introduction is typical for graphene samples irrespective of the number of layers.

The tensile strain decreases the stiffness tensor and increases in lattice anharmonicity^48^. Because long-wavelength phonons mainly contribute to the thermal energy transport, the phonon group velocity for heat conduction is nearly equal to the speed of sound in a material. The speed of sound in a material is proportional to the square root of the stiffness due to the nonlinear character of lattice elasticity. Consequently, the phonon group velocity decreases significantly as the tensile strain increases. Thermal conductivity *κ* is expressed as $\kappa \propto Cv\lambda$, where *C* is the specific heat, *v* is the phonon group velocity, and *λ* is the mean free path (MFP) of a phonon. In other words, the thermal conductivity decreases as the phonon group velocity decreases. In addition, biaxial tensile strain also reduces the stiffness in the out-of-plane direction^48^, drastically changing the thermal conductivity in multilayer graphene compared to that in single-layer graphene.

Next, the change in the phonon MFP due to strain must be considered. According to Matthiessen’s rule, the phonon MFP is described by $\lambda^{ - 1} = \lambda_{ph}^{ - 1} + \lambda_{def}^{ - 1} + \lambda_{GB}^{ - 1} + \lambda_{el}^{ - 1}$, where *λ*_*ph*_*, λ*_*def*_*, λ*_*GB*_*,* and *λ*_*el*_ are the phonon MFP due to intrinsic phonon–phonon, phonon–defect, phonon–grain boundary, and phonon–electron scatterings, respectively. Here, the effect of phonon–phonon scattering must be considered because both DEVICE 1 and DEVICE 2 show identical thermal conductivity changes, implying that other factors may have a relatively small effect.

The strain responsible for thermal conductivity reduction in this study may be nonuniformly distributed over the graphene membrane. Nonuniform strain typically breaks the crystal symmetry of graphene by enhancing phonon–phonon scattering. On the other hand, strain also splits the two degenerate optical dispersion branches (LO and TO) at the G points to create a phonon bandgap^49^. The downshift of phonon frequencies causes more activated phonon modes, enhancing Umklapp scattering. All the aforementioned effects significantly reduce *λ*_*ph*_. Consequently, the decreased phonon MFP drastically reduces the thermal conductivity of the graphene drums.

## Conclusion

Herein we investigated the change in the thermal conductivity of single crystalline multilayer graphene by biaxial strain. Two mechanisms were used to introduce strain: pressure difference–induced strain and electrostatic attraction–induced strain. By precisely estimating the strain using AFM and Raman spectroscopy, we found that both mechanisms drastically reduce the thermal conductivity by 60–70% with a strain of approximately 0.1%. This reduction does not originate from convective heat transfer or phonon–electron scattering, but it may be due to the decreased phonon group velocity and MFP caused by strain. Consequently, strain-controlled heat conduction may realize thermal management devices such as high-efficiency thermal switches and diodes.

## Supplementary Information

Supplementary Figures.
