# Supplementary material for: Controlling the thermal conductivity of multilayer graphene by strain
Source: Sci Rep. 2021 Oct 1;11:19533. doi: 10.1038/s41598-021-98974-x (PMC8486806; doi:10.1038/s41598-021-98974-x)
Supplement: Supplementary file 1 — Supplementary Figures. [file 41598_2021_98974_MOESM1_ESM.pdf]

Supplementary information
for

Controlling the thermal conductivity of multilayer graphene by strain

Kaito Nakagawa[1], Kazuo Satoh[2], Shuichi Murakami[2], Kuniharu Takei[1], Seiji Akita[1], and Takayuki Arie[1,*]

[1]Department of Physics and Electronics, Osaka Prefecture University

1-1 Gakuencho, Naka-ku, Sakai, Osaka 599-8531, Japan

[2]Osaka Research Institute of Industrial Science and Technology

2-7-1 Ayumino, Izumi, Osaka 594-1157, Japan

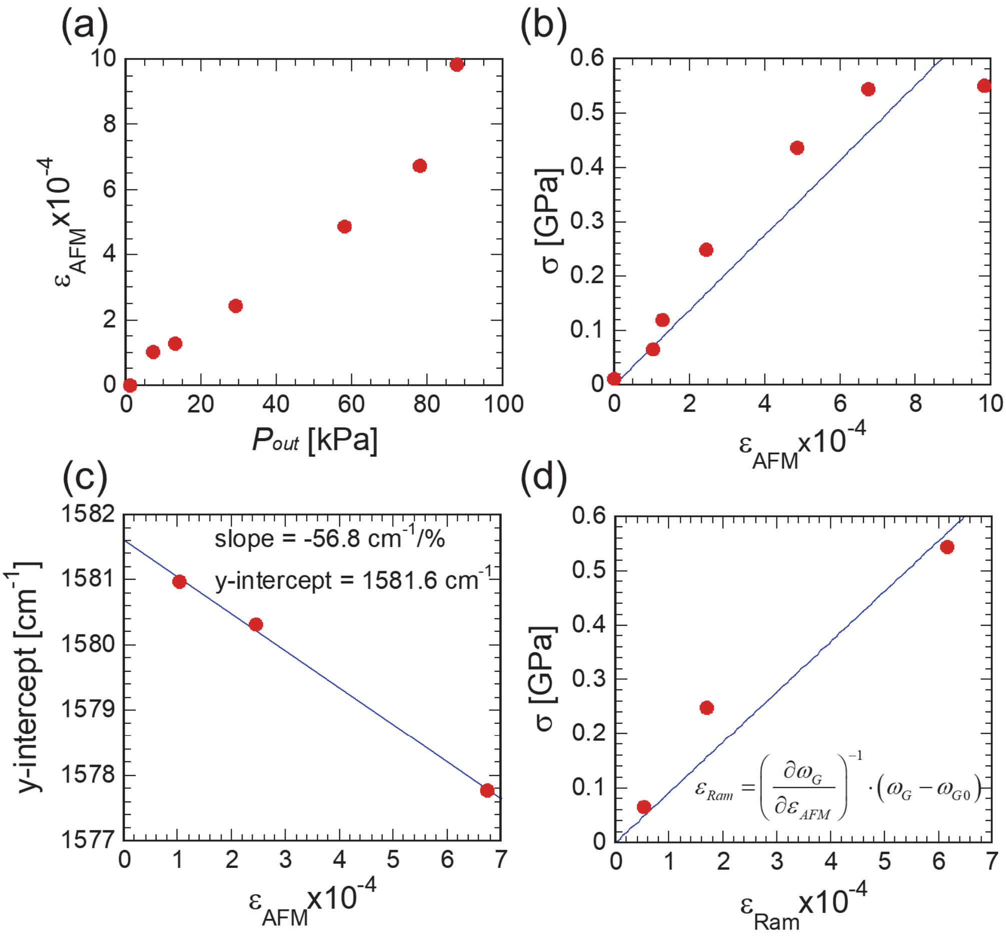

Figure S1. (a) Strain values of DEVICE 1 based on AFM analysis at various pressures P_{out}. (b) Calculated stress–strain curve of the suspended graphene drum extracted from AFM data, giving a mechanical strength of 0.78 TPa. (c) Raman G band peak position without laser heating with respect to the strain estimated based on the AFM analysis, in which two important parameters, $\partial\omega_G/\partial\varepsilon = -56.8\,[cm^{-1}/\%]$ and $\omega_{G0} = 1581.6\,[cm^{-1}]$ are derived. (d) Calculated stress–strain curve of the suspended graphene drum extracted from the Raman spectra, which also gives a mechanical strength of 0.78 TPa.

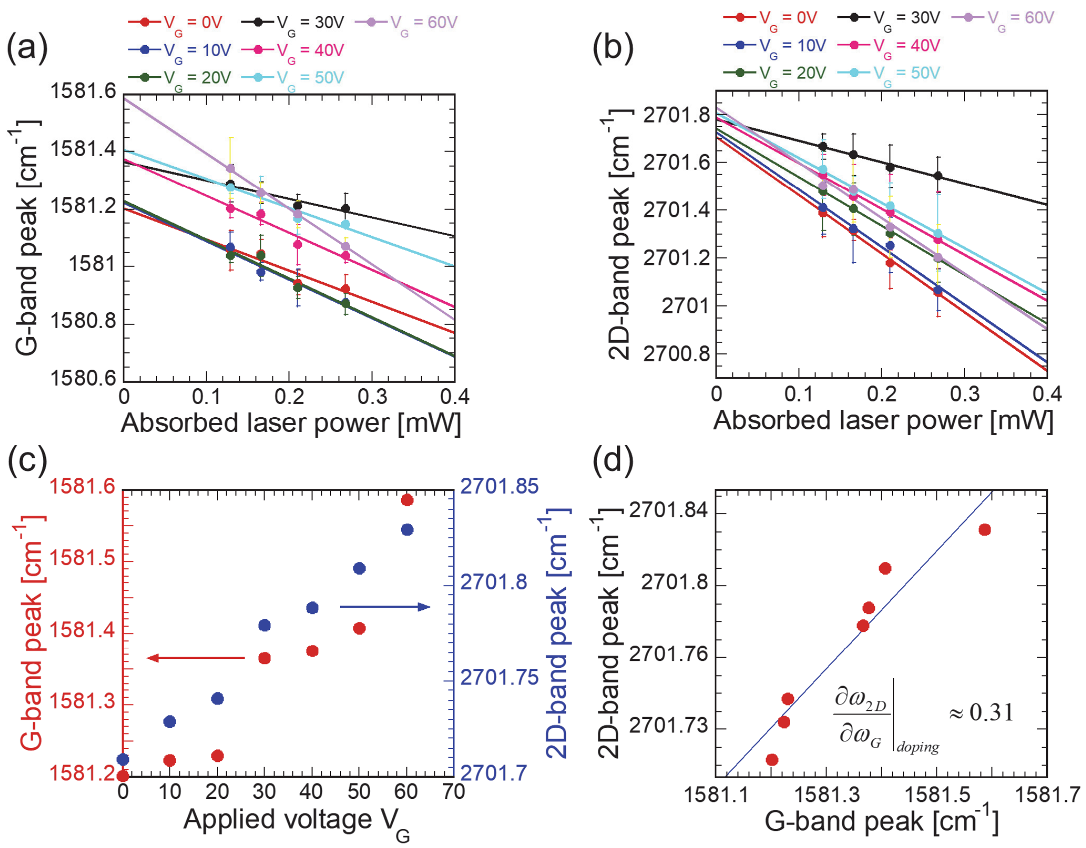

Figure S2. (a) Raman G and (b) 2D band peak positions of the supported region of DEVICE 2 obtained with four different laser powers at various voltages V_G. (c) G and 2D band peak positions without laser heating at various applied voltages V_G. (d) Linear correlation between Raman G and 2D peak positions. Slope indicates a charge doping effect of $\partial\omega_{2D}/\partial\omega_G\big|_{doping} \approx 0.31$.

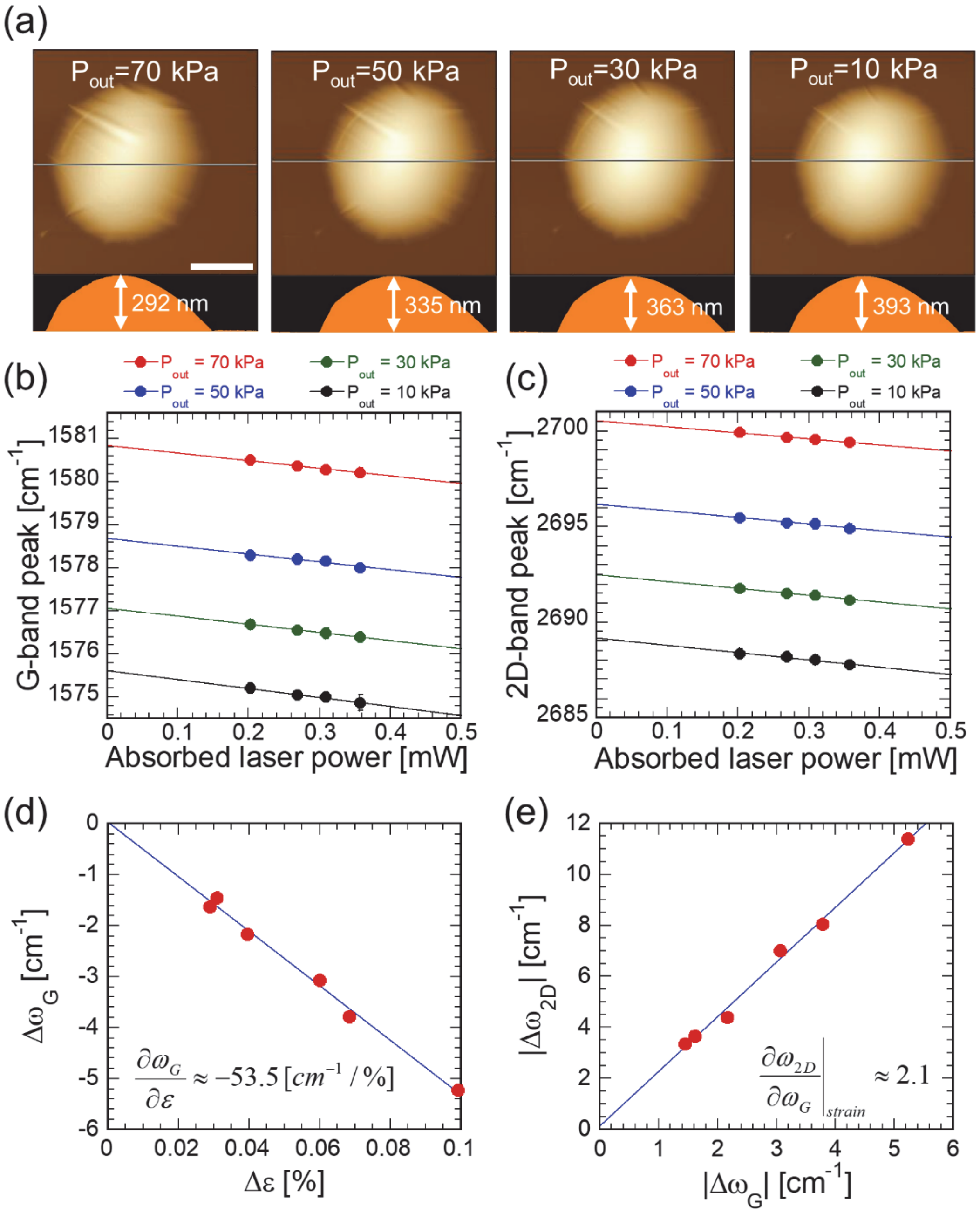

Figure S3. (a) Top and cross-sectional views of the AFM images of the graphene drum to extract the effect of only the strain for DEVICE 2. Bar represents 5μm. (b) Raman G and (c) 2D band peak positions of the graphene drum obtained with four different laser powers at various voltages V_G. (d) Raman G band peak shifts without laser heating with

respect to the strain estimated based on AFM analysis, in which an important parameter, $\partial\omega_G/\partial\varepsilon = -53.5\,[cm^{-1}/\%]$ is derived. (e) Linear correlation between Raman G and 2D peak shifts, which give a slope $\partial\omega_{2D}/\partial\omega_G|_{strain} \approx 2.1$ for the strain effect.

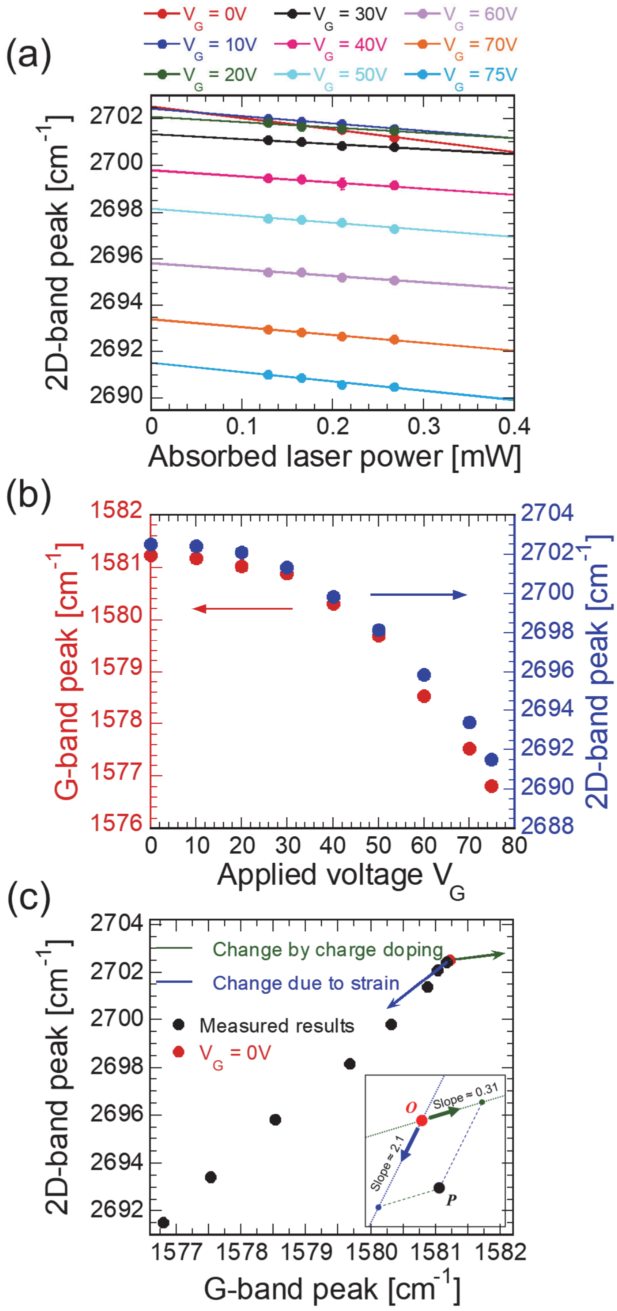

Figure S4. (a) Raman 2D band peak positions of the suspended graphene drum obtained with four different laser powers at various voltages V_G. (b) Raman G and 2D band peak positions without laser heating at various applied voltages V_G obtained by combining

Raman G (Fig. 6 in the main text) and 2D band peak positions. (c) Correlation between Raman G and 2D band positions of the measured results to identify only the strain effect from Raman spectra of the graphene drum for DEVICE 2. Inset shows the decomposition of the exemplified vector ***OP*** with a unit vector and a slope of 0.31 (green arrow for the charge doping effect) and a unit vector with a slope of 2.1 (blue arrow for the strain effect).
